# Tailoring Microstructure and Properties of Nitride Films: Manipulating Bombardment via Regulating Me^+^/Me^2+^ Ratios

**DOI:** 10.3390/nano16120749

**Published:** 2026-06-15

**Authors:** Xingguang Liu, Xin Zhao, Zheng Shu, Yansong Liu, Binhua Gui, Jun Zheng

**Affiliations:** 1 Key Laboratory of Green Fabrication and Surface Technology of Advanced Metal Materials, Anhui University of Technology, Ministry of Education, Ma’anshan 243002, China; 2 Research Center of Laser Fusion, China Academy of Engineering Physics, Mianyang 621900, China; 3 School of Electronic and Information Engineering, Lanzhou Jiaotong University, Lanzhou 730070, China

**Keywords:** HiPIMS, metallic ions, nitride films, Me^+^/Me^2+^ ratios, mechanical properties

## Abstract

Film optimization using high power impulse magnetron sputtering (HiPIMS) currently faces challenges in process control, primarily due to its reliance on empirical trial-and-error adjustment of the macroscopic parameters as well as the insufficient understanding of the underlying mechanisms. To address these issues, this study adopts concentration ratios of monovalent ions over divalent ions of the same metallic element (i.e., Me^+^/Me^2+^) in plasma as a function of key controlled discharge parameters. A mass spectrometer was employed for the in situ diagnostics of ionic species in HiPIMS discharges of Cr, Ti, and Al targets. The influence of discharge parameters on Me^+^/Me^2+^ ratios was systematically investigated. Combined with film characterization, the correlations of discharge parameters, ion concentrations, microstructure evolution, and mechanical properties were established. Results demonstrated that Me^+^/Me^2+^ ratios could be tuned significantly by varying discharge parameters. Decreasing the Me^+^/Me^2+^ ratio suppressed growth of columnar grains and promoted film densification due to enhanced high-energy bombardment. This study reveals the dominant role of the charge state distribution of metallic ions in HiPIMS on the microstructure and properties of nitride films, thereby providing a novel approach to deposition-process optimization, which can also be used as guidance for studies on ternary as well as high-entropy nitride films.

## 1. Introduction

High power impulse magnetron sputtering (HiPIMS), an advanced physical vapor deposition technique developed in the late 1990s, generates high-density plasma with an ionization rate of 70–90% through its unique short-pulse, high peak power discharge mode, far exceeding that of conventional direct current magnetron sputtering (ionization rate < 10%) [1,2,3,4]. This high ionization characteristic significantly increases the proportion of metallic ions among the deposited species, inducing intense ion bombardment on the film-growing surface, which effectively optimizes film density, grain size, and interface bonding. Consequently, HiPIMS demonstrated irreplaceable advantages across various fields, including anti-wear films [5], corrosion-resistant films [6,7], and optical functional films [1,8]. Currently, nitride films, e.g., AlN [9,10], CrN [4,11,12], and TiN [7,13] prepared by HiPIMS are widely applied in critical components, such as engine parts [1,14], precision cutting tools [15,16,17], dies [18,19] and molds [1], which establishes HiPIMS as a core technique for enhancing various tools’ performance.

However, the industrial application of HiPIMS still faces many bottlenecks related to complex process control and unclear underlying mechanisms [1,12,20,21,22,23,24,25]. Current process optimization in both academia and industry often relies on a “black-box” trial-and-error approach based on correlating macroscopic parameters with final film performance. This involves adjusting external parameters such as deposition temperature [26], pulse frequency [20], duty cycle [20], and gas flow ratio [10,27,28], and then observing changes in performance indicators like hardness, friction coefficient, and wear resistance to identify optimal process windows. This paradigm has significant limitations. First, adjusting macroscopic parameters cannot directly reflect changes in the microscopic plasma state, yet microscopic parameters, e.g., charge state distribution and ion energy, act as the core factors determining film growth kinetics. Second, differences in equipment geometry and power supply characteristics lead to vastly different plasma states even under nominally identical macroscopic parameters, resulting in poor process repeatability and difficulties in cross-equipment transfer. This severely restricts the standardization and intelligent development of HiPIMS.

The charge state distribution of metallic ions in the plasma is a key microscopic parameter influencing film growth. According to E = qU (where q is the ion charge number and U is the substrate bias voltage), the bombardment energy carried by divalent ions (M^2+^) is twice that of monovalent ions (Me^+^). With the same bias voltage, the energy directly affects surface migration ability, re-sputtering of deposited atoms, and the generation and recombination efficiency of lattice defects, thereby significantly influencing the film microstructure and macroscopic properties [29]. The bombardment with high-energy M^2+^ suppresses the preferred columnar grain growth effectively, promotes grain refinement and densification, and then enhances film hardness and wear resistance. Furthermore, different elements exhibit inherent differences in ionization energy; the second ionization energy of Cr is 16.5 eV, while that of Ti is only 13.58 eV [24], leading to distinct charge state distribution characteristics under the same HiPIMS discharge conditions. Although previous studies have confirmed the presence of M^2+^ in HiPIMS [3,30,31] and preliminarily explored the influence of individual parameters on ion charge states, existing research still has notable shortcomings. Firstly, the number of investigated systems remains limited [3,21,29,30]. Secondly, there is a disconnect between plasma diagnostics and thin film property studies [3,25,30]; plasma state data have not been quantitatively correlated with the microstructure and mechanical properties of the resulting films, making it difficult to reveal the underlying mechanisms. To address these challenges, this study proposes a new research paradigm centered on the metal ion concentration ratio (Me^+^/Me^2+^) in the plasma as the core control target, aiming to overcome the limitations of the traditional macroscopic parameter trial-and-error approach.

As discussed above, with the utilization of a mass spectrometer for in situ diagnosis of plasma states during HiPIMS discharge of Cr, Ti, and Al targets, we elucidate the regulation pattern of power supply parameters on the Me^+^/Me^2+^ ratio. Combined with characterization techniques such as scanning electron microscopy (SEM), X-ray diffraction (XRD), energy-dispersive X-ray spectroscopy (EDS), and nanoindentation, we establish a quantitative correlation among discharge parameters, plasma state, film microstructure, and film properties, revealing the mechanism by which the ion concentration ratio affects film growth. This study not only provides experimental data to support the understanding of the HiPIMS discharge process and the evolution of film growth and properties but also offers a novel approach for the design and controllable preparation of high-performance nitride films.

## 2. Experimental

### 2.1. Film Preparation

The nitride films were deposited on a single-crystal silicon wafer using a magnetron sputtering film system equipped with a HiPIMS power supply. Prior to deposition, the silicon wafer substrates were sequentially cleaned ultrasonically in acetone and anhydrous ethanol for 10 min and then dried with nitrogen gas. After fixing the samples on a rotating holder, the chamber was evacuated to a base pressure of ≤5.0 × 10^−3^ Pa, and the temperature was set to 180 °C. Before deposition, 180 sccm of argon gas was introduced, and the target was cleaned for 5 min at a power of 500 W. Subsequently, a bias power supply (−1000 V) and an ion source were turned on for 15 min of cleaning to remove residual contaminants and improve film–substrate adhesion. During film deposition, the chamber pressure was maintained at 0.5 Pa, the argon-to-nitrogen flow ratio was 3:1, and the substrate bias voltage was kept at −70 V. Based on preliminary diagnostics, representative parameter sets for the Me^+^/Me^2+^ ratio were selected for film preparation by adjusting the frequency, power, and duty cycle.

### 2.2. Plasma Diagnostics and Film Characterization

A high-sensitivity plasma spectrometer was used for real-time in situ acquisition of ion signals. The average monovalent-to-divalent ion flux ratio (Me^+^/Me^2+^) over a defined period was calculated for each condition. The surface and cross-sectional morphologies of the prepared films were observed using a SU8220 (Hitachi, Tokyo, Japan) cold field emission scanning electron microscope. The elemental composition and distribution were analyzed using an X-Max 20 energy-dispersive X-ray spectrometer (EDS, Oxford Instruments, Oxford, UK) attached to the SEM. The acceleration voltage for both SEM and EDS was fixed at 15 kV. The phase structure of all deposited nitride films was analyzed using an Ultima V X-ray diffractometer (XRD, Rigaku, Tokyo, Japan) with Cu Kα radiation (40 kV, 40 mA). Continuous scanning mode was employed over a 2θ range of 10° to 90° at a scan speed of 10°/min and a step size of 0.01°. Jade software was used for phase analysis and indexing of crystal planes corresponding to the diffraction peaks. The surface chemical composition and elemental chemical states of the CrN-4 film were analyzed using a K-Alpha X-ray photoelectron spectrometer (XPS, Thermo Fisher Scientific, Waltham, MA, USA) with a monochromatic Al Kα X-ray source (hν = 1486.8 eV). The X-ray spot size was 400 μm, and the tube voltage and current were 15 kV and 10 mA, respectively. The base pressure of the analysis chamber was approximately 2 × 10^−9^ mbar. Survey spectra were acquired with a pass energy of 150 eV, a step size of 1 eV, and a dwell time of 100 ms, while high-resolution spectra were collected with a pass energy of 30 eV, a step size of 0.05 eV, five scans, and a dwell time of 300 ms. An electron-ion charge compensation system was used to neutralize surface charging, and the XPS data were processed using Avantage software (Thermo Fisher Scientific, Waltham, MA, USA). Nano-hardness and elastic modulus were measured using a CSM nano-indenter (CSM Instruments, Peseux, Switzerland). At least 10 measurements were performed for each film type, with a spacing of at least 100 µm between adjacent indentations. The average values were calculated to ensure measurement accuracy.

## 3. Results and Discussion

### 3.1. Cr/Al/Ti Ion Concentration Ratios

**Figure 1:**
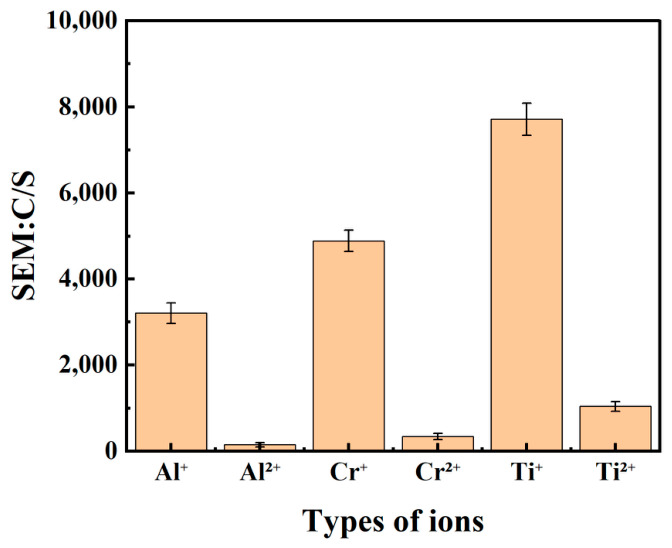
The ion flux of Me^+^ and Me^2+^ with HiPIMS discharge parameters: frequency of 3 kHz, duty cycle of 6%, and power of 4 kW.

During HiPIMS discharge, the ionization behavior of target atoms directly determines the charge state of deposited particles, thereby influencing the microstructure of the film. In this study, a mass spectrometer was used to monitor the ion fluxes (characterized by the signal count rate, SEM) of Al, Cr, and Ti in the sputtering chamber in real time. The analysis focused on the flux characteristics of monovalent ions (Al^+^: *m*/*z* = 27, Cr^+^: *m*/*z* = 52, Ti^+^: *m*/*z* = 48) and divalent ions (Al^2+^: *m*/*z* = 13.5, Cr^2+^: *m*/*z* = 26, Ti^2+^: *m*/*z* = 24) corresponding to the mass-to-charge ratio (*m*/*z*). The regulation mechanisms of power supply frequency, power, and duty cycle on the ion concentration ratio of each element are discussed. Figure 1 shows the temporal ion flux of Al^+^, Al^2+^, Cr^+^, Cr^2+^, Ti^+^ and Ti^2+^ under HiPIMS power supply parameters of 3 kHz frequency, 6% duty cycle, and 4 kW power. The signal of Al^+^ remained at a relatively high level of 6000–8000 counts per second (C/S), while the Al^2+^ signal fluctuated only near the baseline. Under these conditions, the ionization of Al is dominated by monovalent ions, with a low proportion of divalent ions. The SEM signal of Cr^+^ (*m*/*z* = 52) remained at 4500–5500 C/S, while the Cr^2+^ (*m*/*z* = 26) signal was only 0–500 C/S, indicating that the ionization of Cr is also dominated by monovalent ions. The first ionization energy of Cr is 7.44 eV, and the second is 16.50 eV. Although the second ionization energy is lower than that of Al, it is still much higher than the first, so the probability of divalent ionization is low [7]. In addition, the SEM signal of Ti^+^ (*m*/*z* = 48) remained at 7000–8000 C/S, while the Ti^2+^ (*m*/*z* = 24) signal approached 1000 C/S, indicating that the initial ionization of Ti is dominated by monovalent ions. Unlike Al and Cr, the difference between the first (6.82 eV) and second (13.58 eV) ionization energies of Ti is only ~6.76 eV, much smaller than that of Al (~12.85 eV) and Cr (~9.06 eV), providing more easily satisfied energy conditions for divalent ionization.

**Figure 2:**
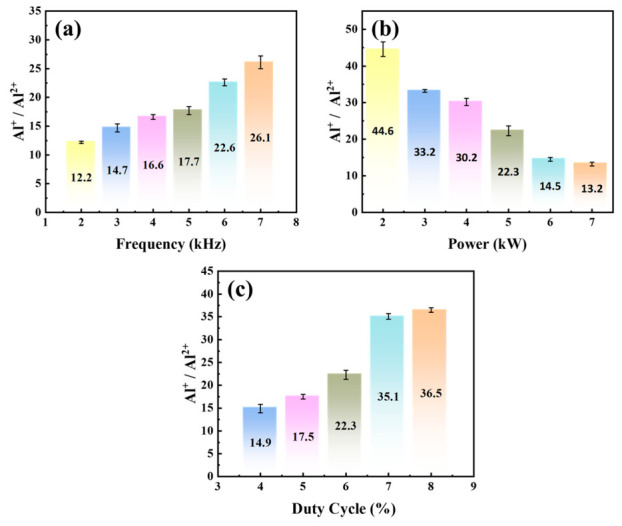
The influence of discharge parameters on Al^+^/Al^2+^ ratios: (**a**) frequency; (**b**) power; (**c**) duty cycle.

Figure 2 shows the influence of power supply frequency, power, and duty cycle on the Al^+^/Al^2+^ concentration ratio. The data points represent the average ratios under multiple different power supply parameter sets, reflecting the statistical regulation pattern. As shown in Figure 2a, when the frequency increased from 2 kHz to 7 kHz, the Al^+^/Al^2+^ ratio increased from 12.2 to 26.1. Under high-frequency pulses, the plasma “refresh” rate increases. Electron energy is more easily maintained near the threshold for monovalent ionization (~6 eV) and struggles to accumulate to the 18.83 eV required for divalent ionization. Therefore, the generation efficiency of monovalent ions increases more significantly. Figure 2b shows the effect of power on Al^+^/Al^2+^. As power increased from 1 kW to 7 kW, Al^+^/Al^2+^ decreased from 44.6 to 12.2. Increasing power enhances the sputtering yield of Al atoms and also raises the electron temperature [30]. Figure 2c shows the effect of duty cycle on Al^+^/Al^2+^. As the duty cycle increased from 3% to 8%, Al^+^/Al^2+^ increased from 14.9 to 36.5. Although longer pulses extend the ionization time, electron energy decreases due to collisional dissipation [32], making it difficult to reach the high-energy threshold for divalent ionization. Consequently, the generation efficiency of divalent ions increases only modestly, leading to a rise in the ratio.

**Figure 3:**
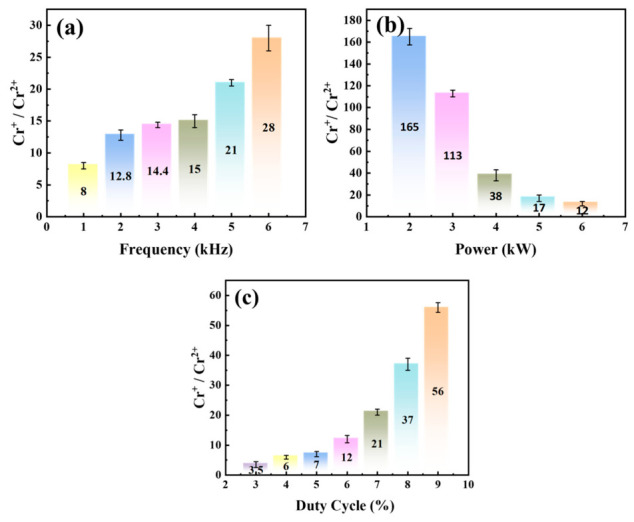
The influence of discharge parameters on Cr^+^/Cr^2+^ ratios: (**a**) frequency; (**b**) power; (**c**) duty cycle.

Figure 3a shows the effect of frequency on Cr^+^/Cr^2+^: as frequency increased from 1 kHz to 6 kHz, Cr^+^/Cr^2+^ increased from 8 to 28. Under high-frequency pulses, electron energy is more easily maintained in the monovalent ionization range, and the high energy required for divalent ionization cannot be accumulated continuously, causing the proportion of divalent ions to decrease relatively. Figure 3b shows the effect of power on Cr^+^/Cr^2+^: As power increased from 1 kW to 6 kW, Cr^+^/Cr^2+^ decreased from 165 to 12. Higher power increases the Cr sputtering yield and electron temperature; the divalent ion flux grows much faster than the monovalent flux, significantly reducing the ratio. Figure 3c shows the effect of duty cycle on Cr^+^/Cr^2+^: as the duty cycle increased from 2% to 9%, Cr^+^/Cr^2+^ increased from 3.5 to 56. Longer pulses extend the collision time between electrons and atoms, allowing more complete monovalent ionization. However, the second ionization energy of Cr (16.50 eV) still requires high electron energy. Under long pulses, electron energy dissipation limits the efficiency of divalent ionization, leading to a substantial increase in the proportion of monovalent ions [33].

**Figure 4:**
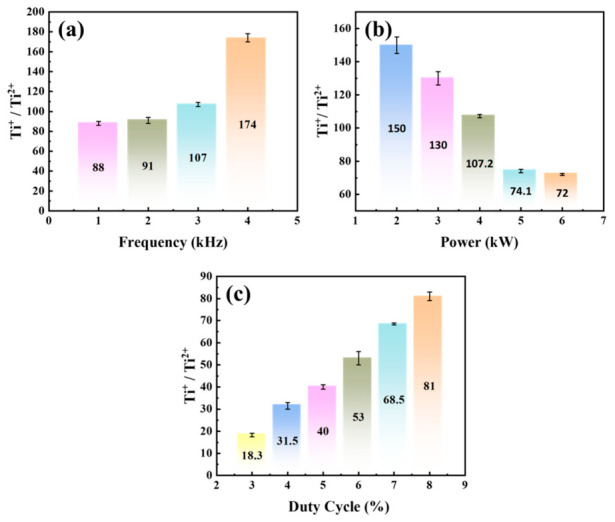
The influence of discharge parameters on Ti^+^/Ti^2+^ ratios: (**a**) frequency; (**b**) power; (**c**) duty cycle.

Figure 4 shows the trend of Ti^+^/Ti^2+^ with power supply parameters, exhibiting the same regulatory trend as Al and Cr. Figure 4c: As the duty cycle increased from 3% to 8%, Ti^+^/Ti^2+^ increased from 18.3 to 81. With longer pulses, electrons have sufficient time to accumulate energy, but the probability of reaching the second ionization energy of Ti (13.58 eV) decreases; simultaneously, plasma density increases, collision frequency between electrons and atoms increases, the probability of divalent ionization decreases, and the ratio rises. Figure 4b: As power increased from 2 kW to 6 kW, Ti^+^/Ti^2+^ decreased from 150 to 72. Higher power increases the electron temperature, causing more electrons to exceed the second ionization energy threshold of Ti. The efficiency of divalent ionization increases faster than that of monovalent ionization, leading to a decrease in the ratio [32]. Figure 4c: As frequency increased from 1 kHz to 4 kHz, Ti^+^/Ti^2+^ increased from 88 to 174. High-frequency pulses maintain a high electron density, making collisions between electrons and atoms more frequent and extending the collision time, allowing more complete monovalent ionization and an increase in the ratio [34].

### 3.2. Nitride Films Prepared Based on Ion Concentration

Based on the plasma state monitoring results, the ion concentration ratio of the target material (Me^+^/Me^2+^) directly determines the charge characteristics of particles during the deposition stage, thereby affecting the film quality and mechanical properties of the resulting nitride films (AlN, CrN, TiN). In this subsection, combining the mass spectrometer measurements with the practical requirements of the HiPIMS process, we first define the reasonable range of Me^+^/Me^2+^ ratios for the three types of nitride films. Subsequently, four typical ratios and corresponding power supply parameters are selected for each elemental target to provide a precise process basis for subsequent experiments.

The range of Me^+^/Me^2+^ ratios must simultaneously satisfy “plasma ionization feasibility” and “film property requirements,” excluding extreme ratios (e.g., Cr^+^/Cr^2+^ = 165 under excessively high power conditions, which can lead to target overheating). With reference to typical application scenarios for nitride films (wear-resistant, corrosion-resistant), a high ratio (Me^+^ dominant) favors higher film density, while a low ratio (increased M^2+^ proportion) enhances particle surface migration ability [34]. The final determined ratio ranges are as follows: AlN films: Al^+^/Al^2+^ ratio range of 15.0–40.0. This range covers common parameter ranges (frequency: 2–6 kHz, power: 2–6 kW, duty cycle: 4–7%), excluding extreme cases such as the baseline ratio of 12.2 (low frequency, low power, insufficient ionization) and 44.6 (high frequency, high power, excessively fast deposition rate) [35]. CrN films: Cr^+^/Cr^2+^ ratio range of 8.0–80.0. Cr exhibits the highest sensitivity in ratio control, so the intermediate stable range is selected, excluding abnormal values such as 3.5 (low duty cycle, low plasma density) and 165 (excessively high power, uneven target sputtering) [36]. TiN films: Ti^+^/Ti^2+^ ratio range of 25.0–120.0. This range covers the common parameter intervals of duty cycle: 3–7% and power: 2–5 kW, excluding extreme conditions such as 18.3 (low duty cycle, excessive divalent ions leading to high film stress) and 174 (low frequency, low power, excessively low proportion of divalent ions).

**Figure 5:**
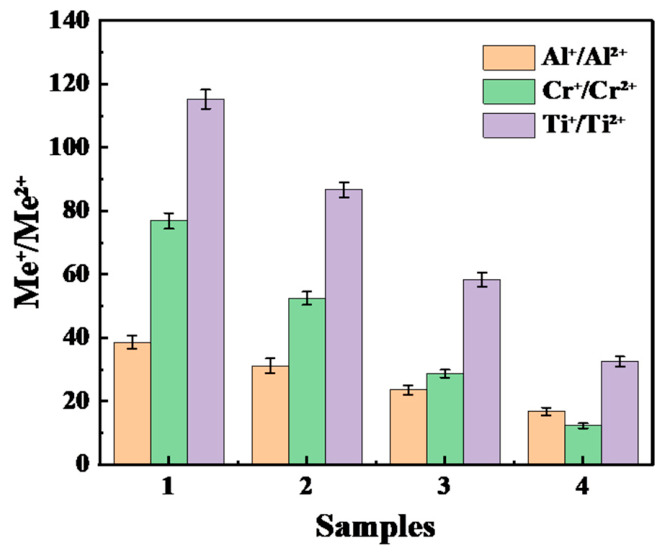
The Me^+^/Me^2+^ ratios selected for depositing AlN, CrN and TiN films.

For the three elemental targets Al, Cr, and Ti, according to the regulation pattern of their Me^+^/Me^2+^ ratio (increasing with higher duty cycle and frequency, decreasing with higher power), four typical ratios (covering low, medium-low, medium-high, and high levels) were selected for each, along with matching HiPIMS power supply parameters and basic process conditions. All parameters were validated by preceding ion monitoring experiments. The parameters for AlN, CrN, and TiN are shown in Table 1 and Figure 5, respectively. Notes: i. Ion ratio precision: All ratio data are presented as the average standard deviation of five consecutive measurements under the corresponding power supply parameters, with a relative error ≤5%, ensuring parameter reliability [37]. ii. Process consistency: The basic conditions for the same elemental target (nitrogen partial pressure, target-substrate distance, deposition temperature) were kept consistent. Only the HiPIMS power supply parameters were adjusted to control the ion ratio, eliminating interference from other factors [38]. iii. Property correlation: The parameter design references previous findings on HiPIMS nitride films: a high Me^+^/Me^2+^ ratio typically corresponds to higher film hardness (due to enhanced ion bombardment), while a low ratio favors lower film internal stress, providing a basis for comparison in subsequent performance tests [39].

The ion concentration ratios of Me^+^/Me^2+^ directly determine the charge state and bombarding energy of the sputtered particles and are one of the core factors regulating the microstructure of films prepared by HiPIMS [40]. This subsection, combined with SEM cross-sectional and surface morphology characterization, analyzes the evolution of the microstructure of AlN, CrN, and TiN films with their respective ion concentration ratios and discusses the underlying physical mechanisms.

### 3.3. Microstructure and Morphologies

**Figure 6:**
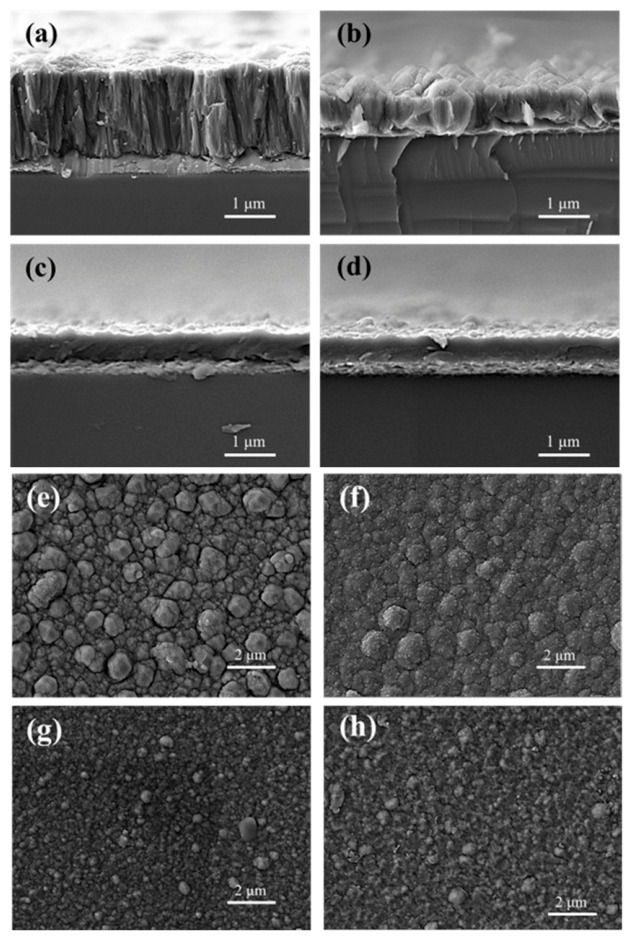
Cross-sectional (**a**–**d**) and surface (**e**–**h**) morphologies of AlN films prepared at different Al^+^/Al^2+^ ratios.

For AlN films, Figure 6a–d shows cross-sectional SEM images at different Al^+^/Al^2+^ ratios. Figure 6a reveals coarse, continuous columnar grains with clear grain boundaries and distinct intergranular gaps. As the ratio decreases, the columnar grains gradually refine, and intergranular bonding becomes tighter. In Figure 6d, the columnar grain characteristics are significantly weakened, and the film cross-section appears more uniform and denser. Figure 6e–h shows surface SEM images of AlN films at different Al^+^/Al^2+^ ratios. Figure 6e shows relatively large surface particles (~500 nm), uneven distribution, and clusters. As the ratio decreases, the particle size gradually reduces. In Figure 6h, the surface particles are refined to approximately 100 nm, and the surface flatness is significantly improved [41]. A decrease in the Al^+^/Al^2+^ ratio corresponds to an increase in the concentration of divalent Al ions. Divalent ions, obtaining twice the charge number than monovalent ions, acquire higher bombardment energy with substrate bias voltage (energy is positively correlated with charge number, E = qU, where U is the substrate bias voltage) [42]. Intense ion bombardment disrupts the preferred growth orientation of columnar grains while promoting surface diffusion and rearrangement of atoms, ultimately achieving grain refinement and film densification [43]. Under high-ratio conditions (monovalent ions dominant), the ion bombardment energy is lower, columnar grain growth is not significantly inhibited, resulting in a typical columnar structure.

**Figure 7:**
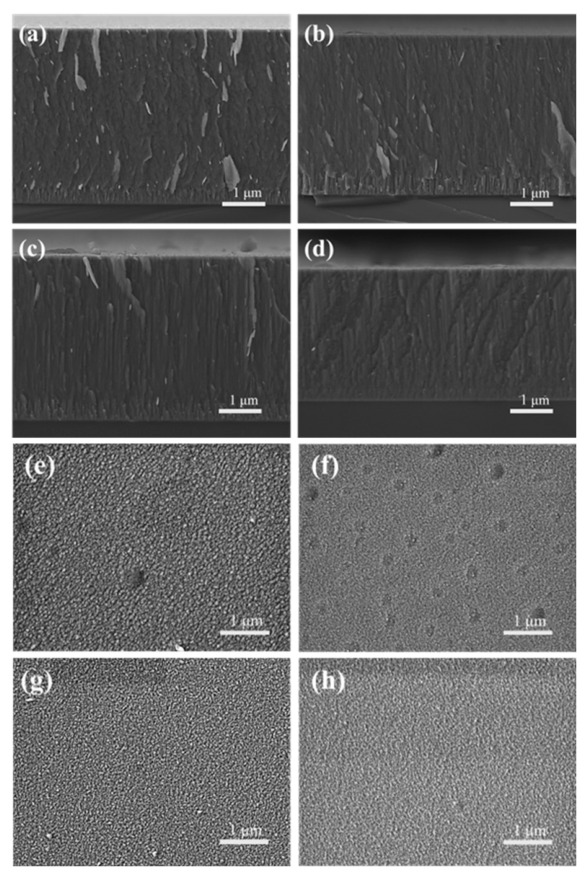
Cross-sectional (**a**–**d**) and surface (**e**–**h**) morphologies of CrN films prepared at different Cr^+^/Cr^2+^ ratios.

For CrN films, Figure 7a–d shows cross-sectional SEM images at different Cr^+^/Cr^2+^ ratios. In Figure 7a, columnar grains are coarse with pronounced orientation and numerous intergranular gaps. As the ratio decreases, the columnar grains rapidly become finer, and intergranular bonding becomes tighter. In Figure 7d, no obvious coarse columnar grains are visible in the cross-section, and the film density is improved.

Figure 7e–h shows surface SEM images of CrN films at different Cr^+^/Cr^2+^ ratios. In Figure 7e, surface particle agglomeration is evident, with a size of approximately 400 nm. As the ratio decreases, particle agglomeration diminishes. In Figure 7h, the surface particles are uniformly refined to approximately 80 nm, and the flatness is greatly enhanced [44]. A decrease in the Cr^+^/Cr^2+^ ratio implies an increase in the proportion of divalent Cr ions. The higher charge of divalent ions induces a stronger ion bombardment effect [34]. Additionally, the atomic mass of Cr is higher than that of Al, resulting in more significant momentum transfer during ion bombardment and a stronger suppression effect on columnar grain growth. This is the primary reason why the structural refinement of CrN films is more pronounced than that of AlN films [45].

**Figure 8:**
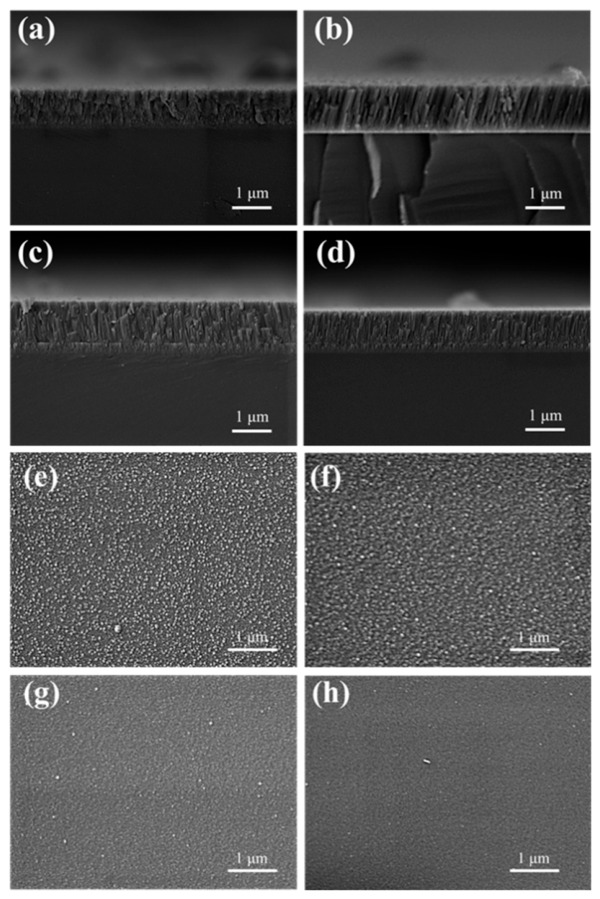
Cross-sectional (**a**–**d**) and surface (**e**–**h**) morphologies of TiN films prepared at different Ti^+^/Ti^2+^ ratios.

For TiN films, Figure 8a–d shows cross-sectional SEM images at different Ti^+^/Ti^2+^ ratios. Figure 8a shows a relatively coarse columnar grain structure. As the ratio decreases, the columnar grains rapidly refine. In Figure 8d, the cross-section exhibits almost no obvious columnar grains, and the film is uniform and dense. Figure 8e–h shows surface SEM images of TiN films at different Ti^+^/Ti^2+^ ratios. In Figure 8e, the surface particle size is approximately 300 nm. As the ratio decreases, the particles continuously refine. In Figure 8h, the surface particle size is only about 50 nm, and the morphology is relatively flat. A decrease in the Ti^+^/Ti^2+^ ratio corresponds to an increase in the power supply parameters, leading not only to an increased proportion of divalent Ti^2+^ ions but also to higher plasma density and electron temperature, significantly increasing both the frequency and energy of ion bombardment [41]. Furthermore, the second ionization energy of Ti (13.58 eV) is much lower than that of Al (18.83 eV) and Cr (16.50 eV) [46], resulting in a more substantial increase in the proportion of divalent ions. The combined effect of intense ion bombardment and high atomic mobility leads to the most significant microstructure refinement among the three nitride films [30].

For all three types of nitride films, as the Me^+^/Me^2+^ ratio decreases, the microstructure shows a consistent trend of “columnar grain refinement—increased density—homogenized surface particles.” The core driving mechanism is the intense ion bombardment effect resulting from the increased proportion of divalent ions. Differences in structural evolution arise from the coupling of inherent target properties and plasma response: i. TiN shows the most significant effect: The proportion of divalent ions increases substantially (small difference in ionization energies), and increasing power supply parameters enhances plasma density and electron temperature. ii. CrN shows an intermediate effect: Cr has a larger atomic mass, leading to more significant momentum transfer during ion bombardment and a stronger suppression effect on columnar grains. iii. AlN shows a relatively mild effect: Al has a small atomic mass, and the increase in the proportion of divalent ions is limited (large difference in ionization energies) [9].

### 3.4. Composition and Phase Analyses

The stoichiometry of a thin film is one of the core parameters determining its physical and chemical properties. During the HiPIMS preparation of nitride films, the concentration distribution of metal ions in the plasma not only affects the growth kinetics and microstructure but also profoundly influences the final chemical combination ratio of metal (M) and nitrogen (N) elements in the film. To elucidate the mechanism by which the Me^+^/Me^2+^ ratio influences the chemical composition, this subsection systematically investigates the variation in the metal and nitrogen atomic percentages (at.%) in AlN, CrN, and TiN films with the ion concentration ratio, aiming to reveal the intrinsic relationship between the plasma state and the reactive sputtering process, thereby providing a theoretical basis for precise control of film stoichiometry.

The phase composition and crystallographic orientation are key factors determining the structural stability of nitride films. During HiPIMS deposition, the ion concentration ratio Me^+^/Me^2+^ can influence the crystal structure characteristics of the film by regulating the arrangement and bonding of atoms [47]. Combined with XRD characterization results, the phase evolution of AlN, CrN, and TiN films, as well as the correlation between the ion concentration ratio and the crystal structure, is analyzed.

Table 2 presents the elemental compositions. Al and N are both uniformly dispersed as fine particles without local enrichment or segregation. The uniformity of the EDS maps shows no significant variation with decreasing Al^+^/Al^2+^ ratio. Although the measured N/Al ratios of the AlN films range from 0.81 to 0.86 (Table 2), they are considered close to the ideal 1:1 stoichiometry, considering the limited sensitivity of EDS to light elements and possible nitrogen deficiency or surface effects. Over the investigated ratio range, the Al content fluctuates narrowly between 53.7 and 55.3 at.%, with the N content correspondingly varying between 44.7 and 46.3 at.%, yielding N/Al ratios between 0.81 and 0.86. These data indicate that the Al^+^/Al^2+^ ratio has a relatively small influence on the stoichiometry of AlN films; the films remain slightly Al-rich but close to stoichiometric under all conditions [9,10].

**Figure 9:**
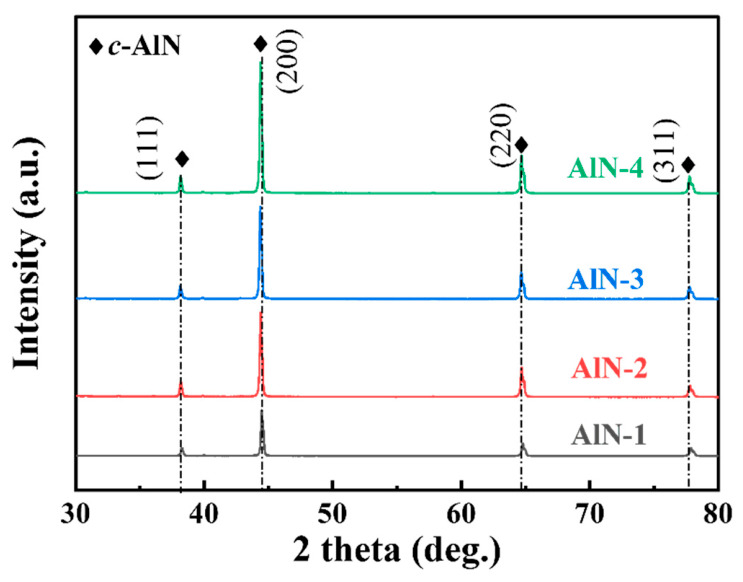
XRD patterns of AlN films prepared at different Al^+^/Al^2+^ ratios.

Figure 9 shows the XRD patterns of AlN films at different Al^+^/Al^2+^ ratios. All diffraction peaks correspond to the characteristic crystal planes ((111), (200), (220), (311)) of cubic AlN (c-AlN). No diffraction peaks from metallic Al or other impurity phases were detected, indicating that a pure cubic AlN phase was formed under all ion concentration ratio conditions. As the Al^+^/Al^2+^ ratio decreases, the intensity of the diffraction peaks gradually increases. Among them, the (200) peak shows the most significant enhancement. Under the high Al^+^/Al^2+^ ratio condition (AlN-1), all peaks are relatively weak and broad. At the low ratio condition (AlN-4), the (200) peak becomes sharp and reaches its maximum intensity. This evolution is directly related to the increased proportion of Al^2+^ ions. A decrease in Al^+^/Al^2+^ implies an increase in the Al^2+^ fraction. The higher charge number of Al^2+^ provides stronger bombardment energy under the substrate bias voltage. This energy transfer promotes the ordered stacking of Al and N atoms during deposition, enhances the crystallinity of c-AlN, and consequently increases the diffraction peak intensities. The gradual dominance of the (200) orientation also originates from the preferred orientation under ion bombardment: the strong bombardment by Al^2+^ favors the growth of the (200) plane, which has a lower surface energy [47]. Strong ion bombardment promotes the ordered arrangement of Al and N atoms along the c-axis direction, increasing the degree of (200) preferred orientation, and thus the peak intensity gradually increases [16].

Table 3 presents the elemental compositions. Cr and N are both uniformly dispersed without obvious segregation. The uniformity of the maps improves slightly for the low-ratio group (d). The data show that all CrN films are Cr-rich, with N contents far below the ideal stoichiometric ratio (50 at.%). Notably, within the higher Cr^+^/Cr^2+^ ratio range (CrN-1 to CrN-3), the N content remains around 30.5 at.%, showing no significant change. Only when the ratio decreases to the lowest condition (CrN-4) does the N content show a relatively clear increase (to 32.73 at.%), raising the N/Cr ratio to 0.49.

**Figure 10:**
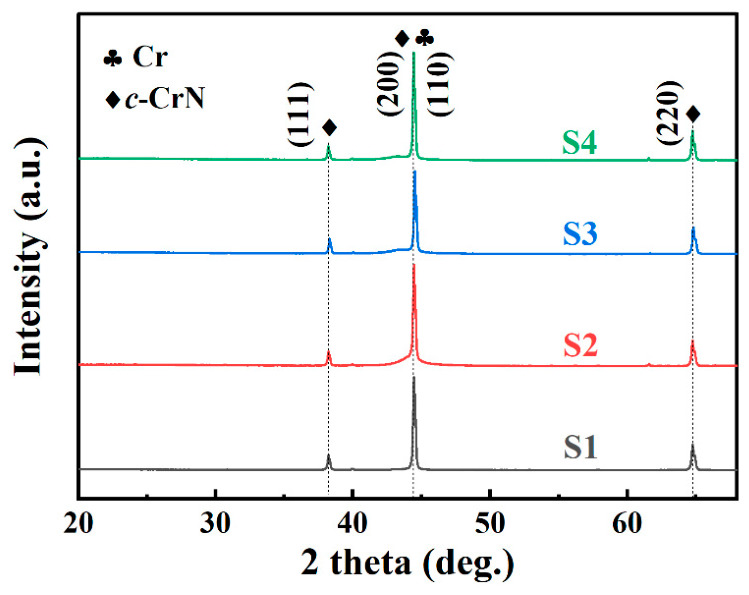
XRD spectra of CrN films prepared at different Cr^+^/Cr^2+^ ratios.

Figure 10 shows the XRD patterns of CrN films at different Cr^+^/Cr^2+^ ratios. The main diffraction peaks can be indexed to the characteristic crystal planes ((111), (200), and (220)) of cubic NaCl-structure CrN, indicating that c-CrN is the dominant crystalline phase in all samples. No diffraction peaks corresponding to Cr_2_N impurities were detected. It should be noted that the diffraction position of metallic Cr overlaps or is very close to the strong c-CrN reflections, especially around the c-CrN (200) peak, and therefore, a small amount of Cr-rich component cannot be completely excluded only by XRD. This is also consistent with the Cr-rich composition revealed by EDS for CrN-4. Nevertheless, the absence of independent Cr_2_N diffraction peaks suggests that nitrogen deficiency is more likely accommodated in the non-stoichiometric CrN_x_ lattice or accompanied by trace Cr-rich regions, rather than forming a detectable Cr_2_N secondary phase. As the Cr^+^/Cr^2+^ ratio decreases, the intensities of the diffraction peaks gradually increase, with the (200) peak showing a particularly pronounced enhancement. Under the high Cr^+^/Cr^2+^ ratio condition (S1), all peaks are relatively weak and broad. At the low ratio condition (CrN-4), the (200) peak reaches its maximum intensity. The core driver of this evolution is the increased proportion of Cr^2+^ ions. The higher charge number of Cr^2+^, combined with the larger atomic mass of Cr, provides stronger bombardment energy and momentum under the substrate bias voltage. This energy transfer promotes the ordered stacking of Cr and N atoms during deposition, directly enhancing the crystallinity of CrN and thereby increasing the diffraction peak intensities [45]. The gradual dominance of the (200) orientation originates from preferred orientation under intense ion bombardment: the strong bombardment by Cr^2+^ suppresses the growth of high-surface-energy crystal planes, favoring the preferred alignment along the (200) plane with lower surface energy [18].

**Figure 11:**
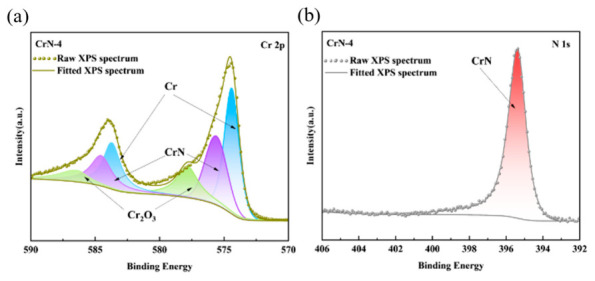
High-resolution XPS spectra of CrN-4 film: (**a**) Cr 2p spectrum and (**b**) N 1s spectrum.

To investigate the relationship between the chemical states of the CrN-4 film and its low N/Cr ratio, XPS analysis was performed. As shown in Figure 11, the high-resolution Cr 2p spectrum can be deconvoluted into three chemical states, namely metallic Cr, Cr–N, and Cr–O. Specifically, the Cr 2p_3/2_ peak at 574.4 eV corresponds to metallic Cr, while the peaks located at 575.5–576.1 eV are assigned to Cr–N bonding [19]. The Cr–O component near 578 eV originates from slight surface oxidation of the sample. The relative area ratio of Cr in the metallic Cr and CrN components is approximately 1:1, indicating that the film consists of nearly equimolar CrN and metallic Cr phases. This result is highly consistent with the N/Cr ratio of 0.49 measured by EDS. However, due to the similar Cr 2p binding energies of CrN and Cr_2_N, the above results can only confirm the existence of metallic Cr, but cannot distinguish between the CrN and Cr_2_N phases. Therefore, identification of the nitride phase requires further analysis based on the N 1s spectrum and XRD results.

The high-resolution N 1s spectrum exhibits a single nearly symmetric main peak at approximately 395 eV, which is assigned to Cr–N bonding, indicating that nitrogen mainly exists in the form of CrN without other nitride chemical states such as Cr_2_N. Furthermore, no Cr_2_N(111) diffraction peak at the theoretical position of approximately 42.5° was observed in the XRD patterns, indicating that no XRD-detectable Cr_2_N crystalline phase was formed in CrN-4. In contrast, the metallic Cr(110) diffraction peak located at approximately 44.3° is close to the c-CrN(200) peak at approximately 43.7°. Considering the peak broadening caused by lattice distortion, microstrain, and grain refinement induced by low-temperature deposition at 180 °C, together with the shift in metallic Cr toward lower 2θ angles due to solid-solution effects and residual stress in Cr-rich regions, these two peaks may overlap within the range of 43.7–44°. Therefore, no independent metallic Cr diffraction peak is observed in the XRD pattern. Combined with the fitting results of the Cr 2p high-resolution spectrum, the coexistence of metallic Cr and CrN phases can be confirmed, with an approximate molar ratio of 1:1.

Combined with the SEM observations, the CrN-4 film exhibits a dense and refined microstructure, which is attributed to the enhanced ion bombardment caused by the high proportion of Cr^2+^ ions, promoting surface migration and atomic rearrangement of deposited species. However, the strong ion bombardment also enhances nitrogen re-sputtering, reducing the effective nitrogen content and resulting in a Cr-rich composition. Overall, the combined EDS, XRD, SEM, and XPS results indicate that the CrN-4 film is mainly composed of a dense non-stoichiometric cubic CrN phase together with metallic Cr components. The relative area ratio of metallic Cr to Cr in the CrN component is approximately 1:1, and the low N/Cr ratio mainly originates from the coexistence of metallic Cr and nitrogen vacancies rather than the formation of a Cr_2_N secondary phase.

Table 4 presents the elemental compositions. Ti and N are uniformly distributed as fine particles. As the Ti^+^/Ti^2+^ ratio decreases, the particle size in the EDS maps refines synchronously (consistent with the surface morphology refinement in Section 3.3), but the uniformity remains stable. All TiN films are Ti-rich (N/Ti < 1). As the Ti^+^/Ti^2+^ ratio decreases from TiN-1 to TiN-4, the nitrogen content does not show a monotonic change. Instead, it reaches its highest value (47.12 at.%) at P2 (corresponding to an intermediate ratio) and shows a distinct low point (42.10 at.%) at P3. This indicates that the control of stoichiometry is not linearly determined by the ion concentration ratio alone but involves a more complex balance of plasma–surface interactions.

**Figure 12:**
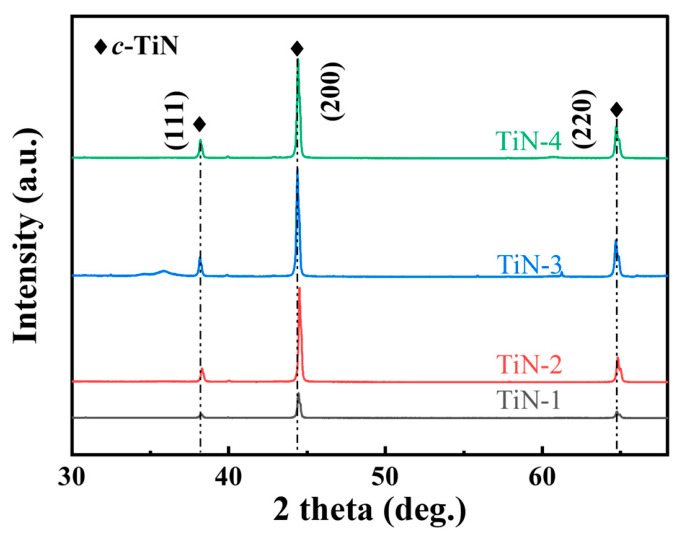
XRD spectra of TiN films prepared at different Ti^+^/Ti^2+^ ratios.

Figure 12 shows the XRD results for TiN films at different Ti^+^/Ti^2+^ ratios. The evolution of the (200) diffraction peak clearly reveals the effect of the Ti^+^/Ti^2+^ ratio on the crystal structure of TiN films. As the samples change from P1 to P4, corresponding to a gradual decrease in Ti^+^/Ti^2+^, the intensity of the (200) diffraction peak shows a systematic increase. The relatively high Ti^+^/Ti^2+^ ratio under condition P1 implies that the deposition process is dominated by lower-energy Ti^+^ ions, which facilitates sufficient atom migration on the growing surface and promotes the formation of preferentially oriented coarse-grained TiN. When the Ti^+^/Ti^2+^ ratio decreases to the P4 condition, the proportion of high-energy Ti^2+^ ions in the plasma increases substantially. Intense ion bombardment not only enhances surface atom mobility, promoting the growth of denser (200)-oriented grains, but also suppresses grain growth through continuous collisions. This ability to regulate the crystalline quality of the (200) plane via the Ti^+^/Ti^2+^ ratio provides a crucial means for the optimization of the mechanical properties of TiN films [48,49].

The high ionization rate characteristic of HiPIMS affects not only metal atoms but also significantly influences the reactive gas (N_2_). High-energy electron collisions can ionize or dissociate N_2_, generating N^+^, N_2_^+^, and active N atoms [50]. Changes in the metal ion charge state, particularly an increase in the proportion of divalent ions, are generally accompanied by an increase in the overall plasma density and electron temperature, which may promote the ionization and activation of nitrogen. The elemental distributions of the three nitride films are characterized by uniform dispersion without obvious segregation, and the fluctuation range of the content is ≤5 at.%. This result is directly related to the effect of the ion concentration ratio [51]. Stoichiometric stability: The chemical composition of AlN films is closest to the 1:1 stoichiometric ratio and is insensitive to changes in the Al^+^/Al^2+^ ratio, which is primarily attributed to the high reactivity and strong bonding energy of Al-N [17]. Metal-rich trend and tunability: CrN films exhibit a Cr-rich state under all conditions. Only when the Cr^+^/Cr^2+^ ratio decreases to its lowest value (~4.0) does the nitrogen content show a relatively clear increase, indicating that achieving stoichiometric CrN requires extremely high plasma ionization and activation conditions. TiN films show a moderately Ti-rich state, and their nitrogen content exhibits a non-linear relationship with the Ti^+^/Ti^2+^ ratio, suggesting the existence of an optimal ion charge state ratio window (approximately 4.9 in this study) for achieving the highest nitrogen incorporation. Core mechanism: The ion charge state ratio influences the final chemical composition of the film primarily by the yield of active nitrogen species in the plasma and the sputtering/implantation balance on the growing surface by high-energy ions. An increase in the proportion of divalent ions, while enhancing the metal ionization rate, also complexly alters the plasma environment and the film–surface interaction. The final stoichiometry is the result of competition and synergy among these factors [52]. This subsection clarifies the possibility and complexity of optimizing the stoichiometry of nitride films by the ion charge state ratio in HiPIMS, especially for TiN and CrN systems, where fine balancing of process parameters is required to achieve the desired chemical composition and properties.

### 3.5. Mechanical Properties

The mechanical properties of the as-deposited films, particularly hardness and elastic modulus, are key indicators for their application as protective films or functional layers. These properties fundamentally depend on the chemical composition, bonding type, crystal structure, and microstructural characteristics of the film. Previous sections have confirmed that during HiPIMS, the metal ion concentration ratio (Me^+^/Me^2+^) by power supply parameters significantly affects the microstructural density and chemical composition of AlN, CrN, and TiN films. This subsection aims to further explore the influence of this ion concentration ratio on the ultimate mechanical properties of the films [53]. The hardness and elastic modulus of the films prepared under different Me^+^/Me^2+^ ratios were systematically measured using nanoindentation. Combined with the structural and compositional analyses from previous sections, the underlying mechanisms of the mechanical property evolution are elucidated.

**Figure 13:**
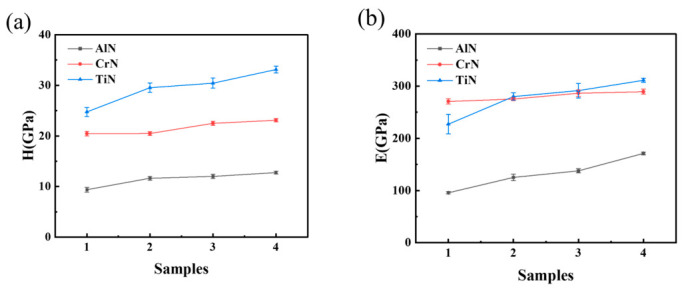
The hardness and elastic modulus of AlN, TiN and CrN films prepared at different Me^+^/Me^2+^ ratios. (**a**) Hardness, (**b**) elastic modulus.

Figure 13 shows the hardness and elastic modulus of AlN films at different Me^+^/Me^2+^ ratios. The hardness of the AlN films steadily increases as the ion concentration ratio decreases. The hardness of AlN-1 is approximately 9 GPa, while that of AlN-4 reaches approximately 12 GPa. The elastic modulus increases from approximately 90 GPa for AlN-1 to approximately 170 GPa for AlN-4. The H-E correlation plot further indicates a clear positive correlation between hardness and elastic modulus. Although the initial hardness of AlN (AlN-1) is higher than that of CrN (CrN-1), its absolute increase with changing ion ratio is comparable to that of the CrN system, with a relative increase of approximately 80%. The hardness of the CrN films increases monotonically and significantly as the Cr^+^/Cr^2+^ ratio decreases. The hardness of CrN-1 (high ratio ~76.8) is approximately 20 GPa, while that of CrN-4 (low ratio ~12.3) increases to approximately 24 GPa. The elastic modulus exhibits a similar but slower increasing trend compared to hardness. The elastic modulus of CrN-1 is approximately 270 GPa, increasing to above 280 GPa for CrN4. High hardness is generally associated with good wear resistance, while the H/E ratio (elastic strain resistance) and the H^3^/E^2^ ratio (resistance to plastic deformation) are better indicators for evaluating toughness and wear resistance [54]. The CrN-4 sample, with its highest hardness and moderate modulus increase, is expected to possess the optimal H/E and H^3^/E^2^ values. As for TiN films at different Ti^+^/Ti^2+^ ratios, as the Ti^+^/Ti^2+^ ratio decreases, both the hardness (H) and elastic modulus (E) of the films show a continuous increasing trend. Under the high Ti^+^/Ti^2+^ ratio condition (TiN-1), the film hardness is approximately 25 GPa, and the elastic modulus is approximately 250 GPa. Under the low Ti^+^/Ti^2+^ ratio condition (TiN-4), the hardness increases to approximately 33 GPa and the elastic modulus approaches 330 GPa, representing increases of 32% for both properties. A decrease in Ti^+^/Ti^2+^ implies a significant increase in the proportion of Ti^2+^ ions. The atomic mass of Ti (48 u) and the high charge number of Ti^2+^ result in much higher bombardment energy (E = qU) and momentum (*p* = mv) under the substrate bias voltage compared to Ti^+^ [55]. This intense ion bombardment not only disrupts the preferred growth orientation of columnar grains, refining the grain size from several hundred nanometers for TiN-1 to several tens of nanometers for TiN-4, but also increases film density and reduces intergranular defects. The hardness of dense TiN films prepared by HiPIMS reported in the literature is generally higher than that of films deposited by DC magnetron sputtering [56]. The relatively large atomic mass of Ti allows for more effective momentum transfer by high-energy Ti^2+^ ions, resulting in more significant densification and strengthening effects.

The differences in mechanical properties of the three films can essentially be attributed to the coupling of the film’s bond energy, atomic mass, and the degree of microstructure refinement. CrN exhibits the highest initial performance due to its high bond energy, and TiN shows the largest performance improvement due to its most significant microstructural refinement; meanwhile, AlN demonstrates stable behavior in the low-to-medium performance range. As the Me^+^/Me^2+^ ratio decreases, the film’s microstructure evolves from porous columnar grains to a dense nanocrystalline structure. Defects such as pores and columnar grain boundaries are mechanically weak points in the film, prone to crack initiation and propagation under load. The intense ion bombardment brought about by a high proportion of divalent ions in the HiPIMS process effectively eliminates these defects by enhancing surface atom mobility and inducing atomic-scale mixing, thereby significantly improving the load-bearing capacity and hardness of the film [57]. According to the Hall–Petch relationship, the hardness of a material increases with decreasing grain size. High-energy ion bombardment not only suppresses grain growth but also refines grains by increasing the nucleation density. Although EDS surface analysis did not reveal drastic changes in stoichiometry, the films prepared under low Me^+^/Me^2+^ ratio conditions (e.g., CrN-4, AlN-4) exhibit nanocrystalline features in their SEM surface morphologies (grain size < 20 nm). This nanostructure is a significant contributing factor to the hardness increase [58]. Combining the mechanical property data with the structural and compositional data, the process optimization windows for different application targets can be defined: i. High hardness/wear-resistant films: Low Me^+^/Me^2+^ ratio conditions should be selected to maximize the densification and strengthening effects of divalent ions, achieving the highest hardness and H^3^/E^2^ ratio. ii. High toughness/impact-resistant films: Excessively high hardness and residual stress may lead to brittleness. Moderate-to-low Me^+^/Me^2+^ ratio conditions may provide a relatively good toughness balance while maintaining reasonably high hardness. iii. Low stress/thickness films: For applications requiring low stress or deposition of thick films, high Me^+^/Me^2+^ ratio conditions, despite yielding lower hardness, result in lower internal stress and reduced risk of delamination.

## 4. Conclusions

In order to correlate HiPIMS discharge parameters with the microstructure evolution and mechanical properties of nitride films, Me^+^/Me^2+^ ratios (i.e., Al^+^/Al^2+^, Cr^+^/Cr^2+^, and Ti^+^/Ti^2+^) were adopted as a novel regulating parameter, which was determined by in situ diagnostics of ionic species using mass spectrometry. Conclusions as follows can be drawn:

1. With the pulse frequency and duty cycle increased, the Al^+^/Al^2+^, Cr^+^/Cr^2+^, and Ti^+^/Ti^2+^ ratios all increased. Conversely, as the power increases, these ratios decrease significantly. This phenomenon should be ascribed to the differences in the ionization energies of the Al, Cr and Ti. Among them, Ti, with the lowest second ionization energy, exhibits the widest range of variation in its Ti^+^/Ti^2+^ ratio in response to power supply parameter adjustments.

2. As the Me^+^/Me^2+^ ratio decreases, the cross-sectional structures of AlN, CrN, and TiN films evolve from coarse columnar grains to fine nanocrystalline or dense featureless structures. Meanwhile, the surface morphologies also evolved from rough and granular to smooth and uniform. AlN films exhibited a near-stoichiometric composition and a single cubic phase across all investigated ratios. CrN films were generally Cr-rich, with the nitrogen content increased slightly. XRD analysis shows that the crystallinity of the films was enhanced with the obvious (200) preferred orientation. This structural refinement and densification primarily originate from the higher bombardment energy carried by divalent ions, which effectively promotes the surface migration of deposited atoms and suppresses columnar grain growth.

3. Nanoindentation tests demonstrated that the hardness and elastic modulus of AlN, CrN, and TiN films increased monotonically as the Me^+^/Me^2+^ ratio decreased. Among them, TiN films exhibited the largest increase in hardness (from 25 GPa to 33 GPa) due to the most significant structural refinement. The performance enhancement was primarily attributed to film densification, grain refinement, and defect reduction. TiN exhibited the optimal structure refinement and performance enhancement because of the easier adjustment of Ti^+^/Ti^2+^ ratios.

This study has revealed and proved the strong relationship between the Me^+^/Me^2+^ ratio and the microstructure/properties of HiPIMS nitride films, proving a novel and effective approach for film optimization of similar binary systems, as well as more complicated systems such as ternary or high-entropy nitrides.

## Figures and Tables

**Table 1:** The corresponding nitride films prepared parameters.

Group	Frequency (kHz)	Power (kW)	Duty Cycle (%)
AlN-1	2	3	8
AlN-2	4	1	5
AlN-3	2	3	5
AlN-4	2	5	5
CrN-1	2	3	5
CrN-2	4	4	5
CrN-3	2	4	8
CrN-4	2	5	5
TiN-1	2	2	5
TiN-2	2	4	8
TiN-3	4	4	5
TiN-4	2	6	5

**Table 2:** Elemental analysis of AlN films prepared at different Al^+^/Al^2+^ ratios.

Samples	Elemental Composition (at.%)		
	Al	N	N/Al Ratio
AlN-1	53.70	46.30	0.86
AlN-2	54.30	45.70	0.84
AlN-3	55.31	44.69	0.81
AlN-4	54.61	45.39	0.83

**Table 3:** Elemental analysis of CrN films prepared at different Cr^+^/Cr^2+^ ratios.

Samples	Elemental Composition (at.%)		
	Cr	N	N/Cr Ratio
CrN-1	69.48	30.52	0.44
CrN-2	70.15	29.85	0.43
CrN-3	69.90	30.40	0.43
CrN-4	67.27	32.73	0.49

**Table 4:** Elemental analysis of TiN Films prepared at different Ti^+^/Ti^2+^ ratios.

Samples	Elemental Composition (at.%)		
	Ti	N	N/Ti Ratio
TiN-1	55.04	44.96	0.82
TiN-2	52.88	47.12	0.89
TiN-3	57.90	42.10	0.73
TiN-4	53.93	46.07	0.85

## Data Availability

The original contributions presented in this study are included in the article. Further inquiries can be directed to the corresponding authors.
